# Supplementary material for: IFT proteins spatially control the geometry of cleavage furrow ingression and lumen positioning
Source: Nat Commun. 2017 Dec 4;8:1928. doi: 10.1038/s41467-017-01479-3 (PMC5715026; doi:10.1038/s41467-017-01479-3)
Supplement: Supplementary file 1 — Supplementary Information [file 41467_2017_1479_MOESM1_ESM.pdf]

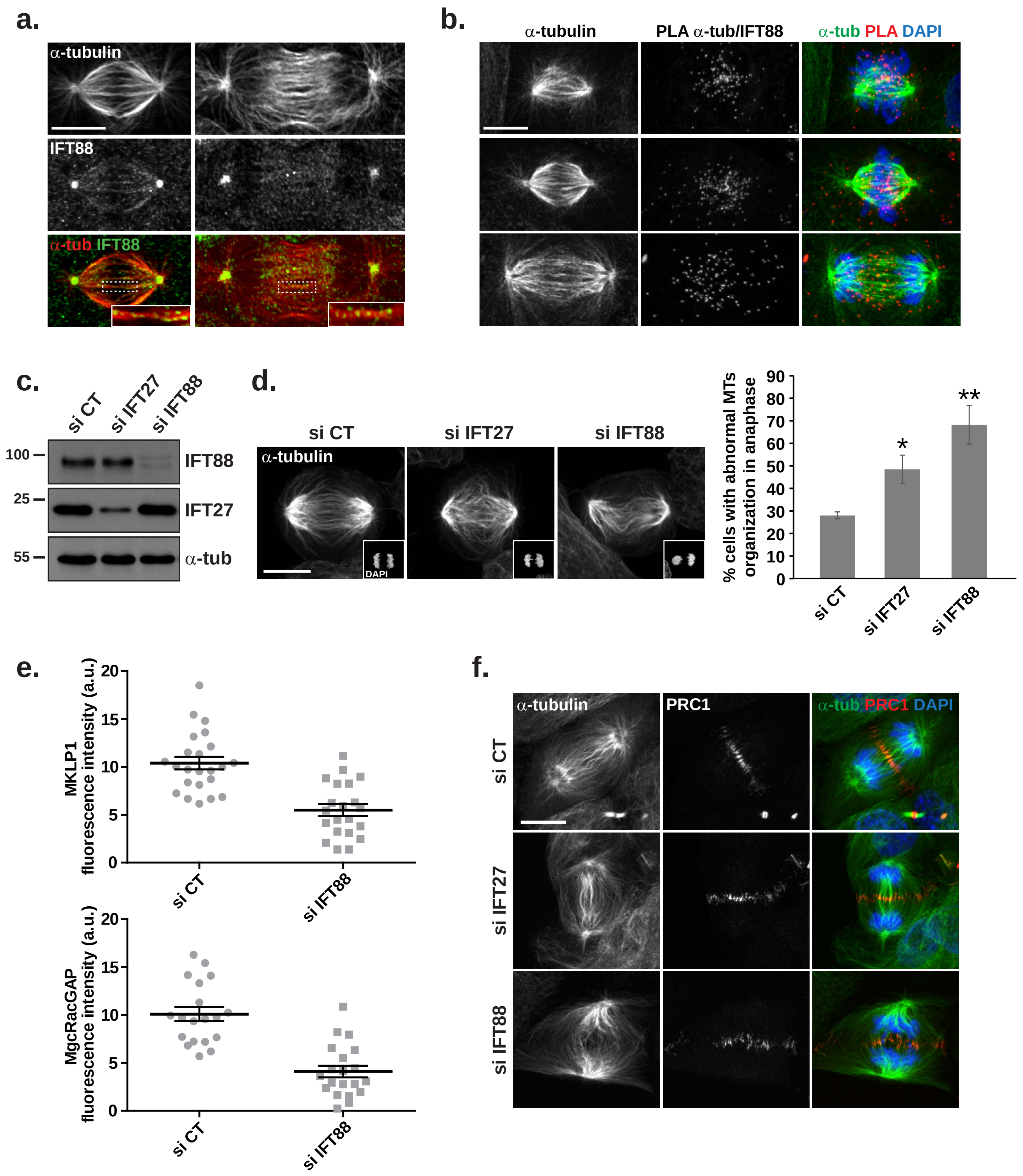

Supplementary Figure 1. IFT proteins are required for central spindle MTs organization.
(a) Immunofluorescence images showing endogenous IFT88 localization in metaphase and anaphase LLC-PK1 cells. Insets, IFT88 staining showing dots along the length of MTs. **(b)** PLA showing the association between α-tubulin and IFT88 in prometaphase, metaphase and anaphase LLC-PK1 cells. Maximum projections of α-tubulin (α-tubulin-FITC) and PLA staining are shown. **(c)** Western blots showing the amount of IFT27 or IFT88 in HeLa Kyoto cells transfected with CT, IFT27 or IFT88 siRNA. α-tubulin: loading control. **(d)** Immunofluorescence images (left) showing α-tubulin staining in anaphase HeLa Kyoto cells transfected with CT, IFT27 or IFT88 siRNA show abnormal central spindle MTs organization in IFT-depleted cells. DAPI (inset). Quantification (right) of the percentage of anaphase HeLa Kyoto cells with abnormal central spindle MTs organization. n>150 cells for each condition. 3 independent experiments. Mean +/- s.d. *, p<0.05 and **, p<0.01 compared to control (t-test). **(e)** Quantification of MKLP1 (top) and MgcRacGAP (bottom) fluorescence intensity at the central spindle. n>19 cells for each condition. Mean +/- s.e.m. p<0.001 compared to control (t-test). **(f)** Immunofluorescence images showing PRC1 localization in anaphase LLC-PK1 cells transfected with CT, IFT27 or IFT88 siRNA. Images show that PRC1 still localizes to the central spindle upon IFT proteins depletion. In all panels, scale bars: 10 μm.

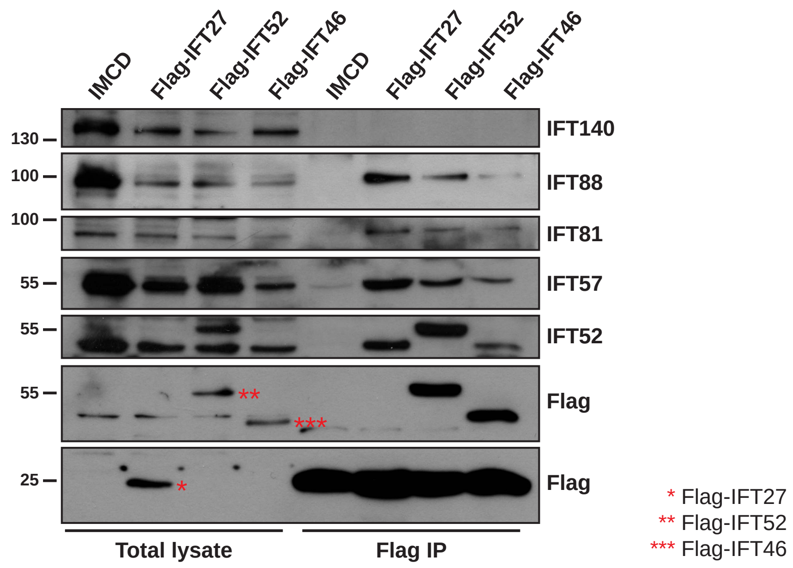

Supplementary Figure 2. A subset of IFT-B proteins interact in mitosis.
Flag immunoprecipitations performed on mitotic shake-off of IMCD cells stably expressing or not Flag-tagged IFT27, IFT52 or IFT46 showing that IFT-B proteins interact in mitosis. Immunoblots were performed as indicated with antibodies against IFT proteins or Flag. Asterisks show the expression levels of Flag-tagged IFT proteins.

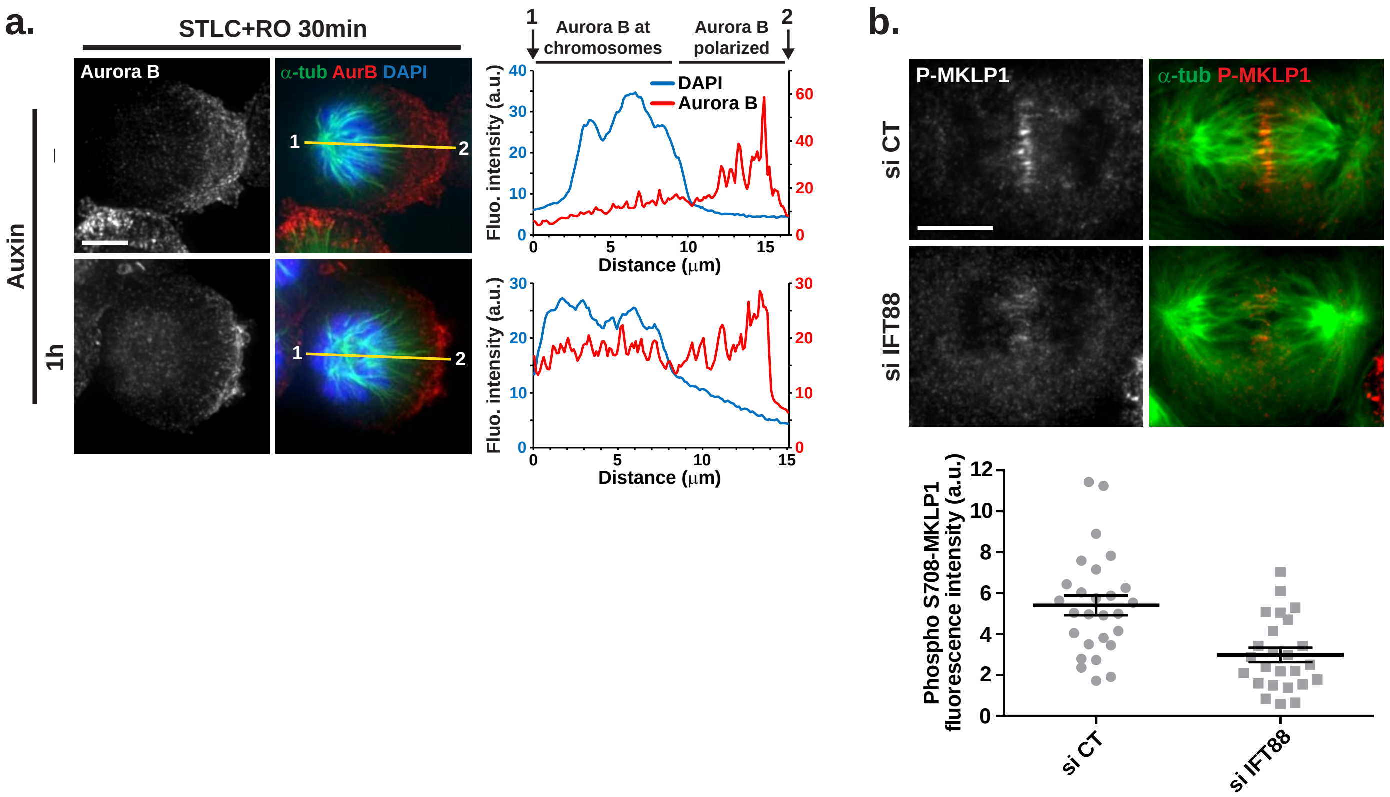

Supplementary Figure 3. Aurora B relocalization is impaired and Phospho S708-MKLP1 is decreased at the central spindle upon IFT88 depletion.
(a) Immunofluorescence images (left) showing Aurora B staining (red) in HCT116 AID-IFT88 cells undergoing monopolar cytokinesis (STLC+RO3306 30 min). Control (-) and Auxin (1h)-induced AID-YFP-IFT88 degradation conditions are shown. α-tubulin (green) and DAPI (blue) are used to monitor cell polarization. Scale bar: 5 μm. Line scans (right) indicate whether Aurora B is well polarized or delayed. Aurora B and DAPI fluorescence intensities are measured from 1 to 2 along the yellow line shown in the α-tubulin/Aurora B/DAPI immunofluorescence images. **(b)** Immunofluorescence images (top) showing Phospho S708-MKLP1 (P-MKLP1) and α-tubulin stainings in anaphase LLC-PK1 cells. Scale bar: 10 μm. Quantification (bottom) of Phospho S708-MKLP1 fluorescence intensity at the central spindle. n>25 cells for each condition. Mean +/- s.e.m. $p<0.001$ compared to control (t-test).

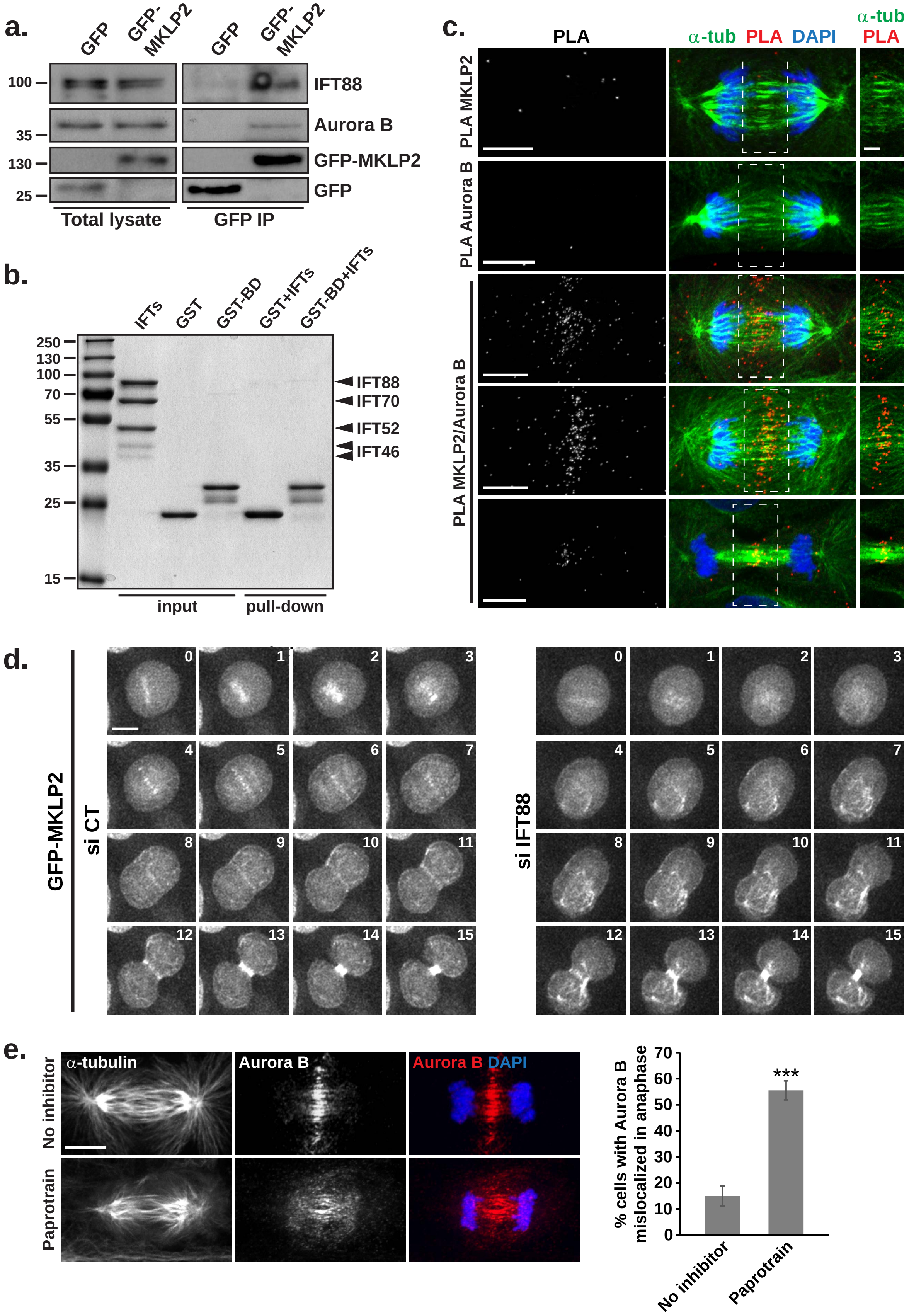

Supplementary Figure 4. MKLP2 interacts with IFT88 and is required for Aurora B relocalization in anaphase. **(a)** GFP immunoprecipitations performed on mitotic GFP or GFP-MKLP2 HeLa Kyoto cells show an interaction between GFP-MKLP2, IFT88 and Aurora B. **(b)** Coomassie blue stained gel showing recombinant proteins used for pull-down assays (input) and results of pull-down assays. GST (negative control); GST-BD (IFT88 binding domain of MKLP2); IFTs: sub-complex of IFT88+IFT70+IFT52+IFT46. **(c)** PLA showing the association between MKLP2 and Aurora B in anaphase and at late stage of cytokinesis in LLC-PK1 cells. MKLP2 and Aurora B antibodies were used alone as negative controls. Maximum projections are shown. Inset, right: single confocal section of the dashed boxes area. **(d)** Images from time-lapse microscopy of mitotic HeLa Kyoto cells expressing GFP-MKLP2 and transfected with control or IFT88 siRNA. Time (min). **(e)** Immunofluorescence (left) showing α-tubulin and Aurora B stainings in LLC-PK1 cells treated or not with Paprotrain (5µM). Quantification (right) of the percentage of cells with Aurora B mislocalization in anaphase. n>70 cells for each condition. 3 independent experiments. Mean +/- s.d. ***, $p<0.001$ compared to non-treated condition (t-test). In all panels, scale bars: 10 µm for main images, 3 µm for insets.

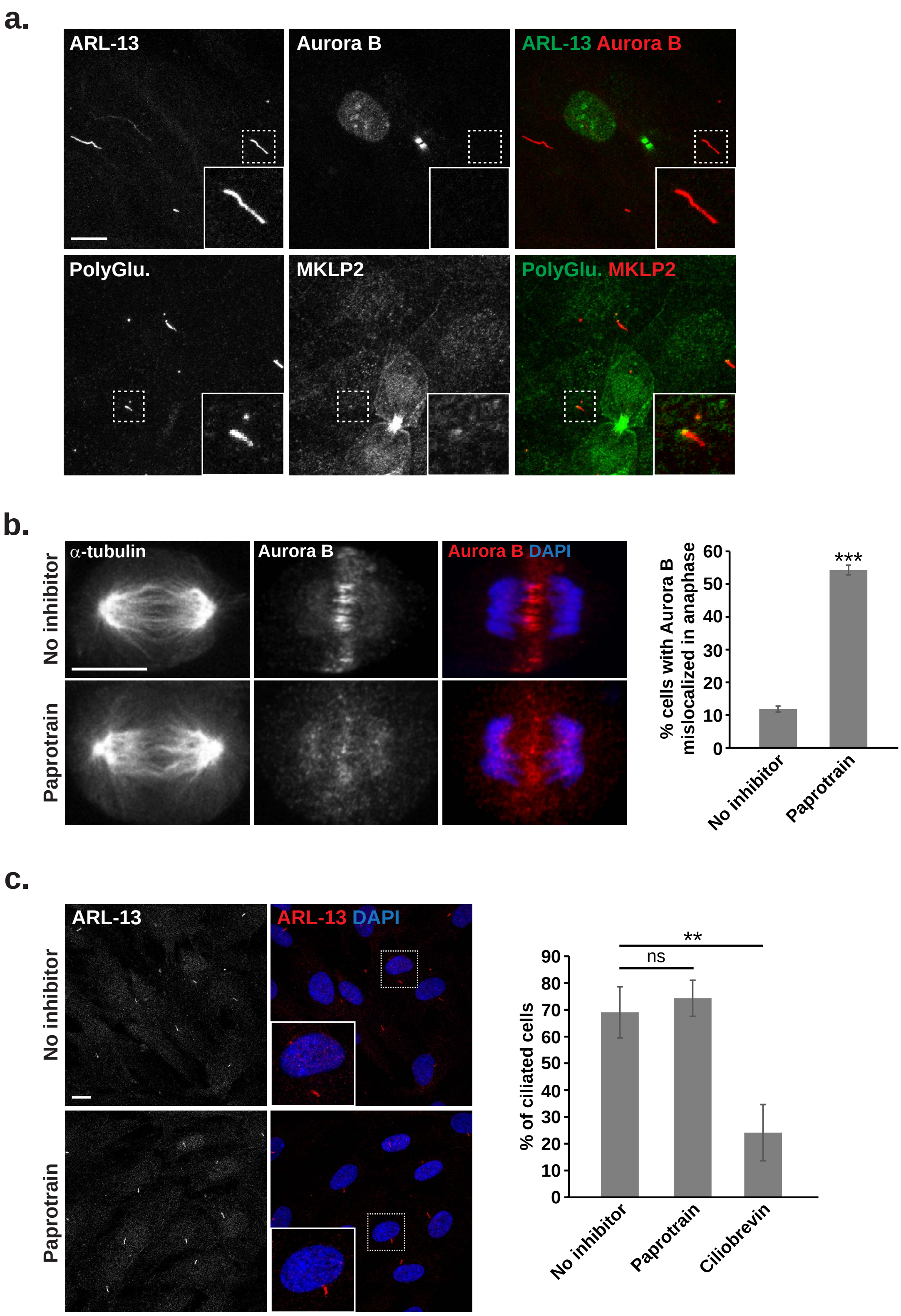

Supplementary Figure 5. Low dose of MKLP2 inhibitor does not affect cilia formation.
(a) Immunofluorescence images showing the localization of Aurora B and MKLP2 in ciliated LLC-PK1 cells. ARL-13 and Polyglutamylated Tubulin are used as cilia markers. Insets: magnified dashed boxes regions. MKLP2 staining is observed at the base of cilia whereas Aurora B is not, indicating that the MKLP2/Aurora B complex is not present at the ciliary base. **(b)** Immunofluorescence (left) showing α-tubulin and Aurora B stainings in RPE cells treated or not with Paprotrain (5μM). Quantification (right) of the percentage of cells with Aurora B mislocalization in anaphase. n>52 cells for each condition. 3 independent experiments. Mean +/- s.d. ***, $p<0.001$ compared to no inhibitor condition (t-test). **(c)** Immunofluorescence (left) showing ARL-13 staining (red) to monitor cilia formation in RPE cells treated or not with Paprotrain. Inset: magnification of the boxed area. Quantification (right) of the percentage of ciliated RPE cells. Ciliobrevin D was used as inhibitor of cilia formation. n>220 cells for each condition. 3 independent experiments. Mean +/- s.d. ns: not significant and **, $p<0.01$ compared to no inhibitor condition (t-test). In all panels, scale bars: 10 μm.

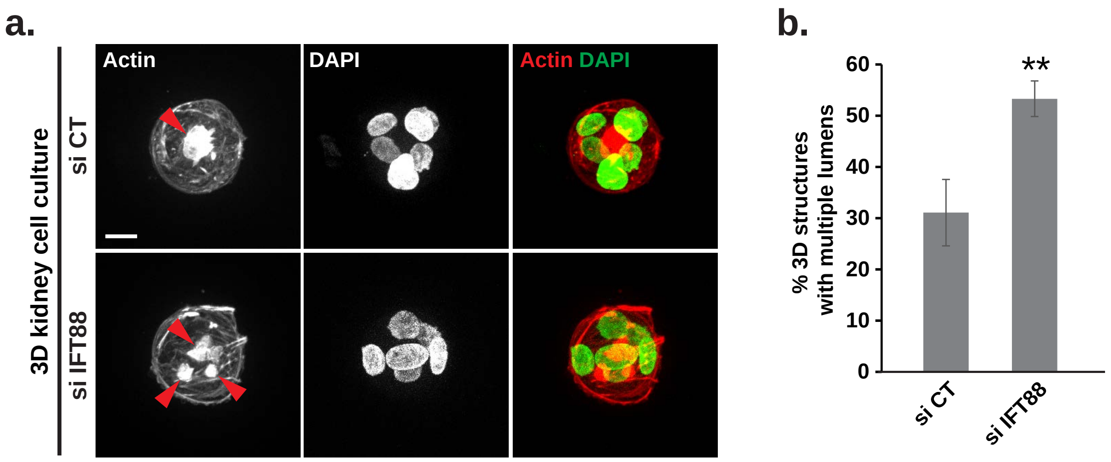

Supplementary Figure 6. IFT88 is required for the formation of a solitary central lumen in 3D renal cultures.
(a) LLC-PK1 cells depleted or not in IFT88 were grown in 3D on disk-shaped micropatterns for 48h to form 3D structures. Images show 6-cells (DAPI staining) structures in which lumens (red arrows) are detected with actin staining. A solitary central lumen is observed in control condition whereas multifocal lumens are observed upon IFT88 depletion. Scale bar: 10 µm. **(b)** Quantification of 3-6 cells stage 3D structures of LLC-PK1 with multiple lumens. n>70 for each condition. 3 independent experiments. Mean +/- s.d. **, p<0.01 compared to control (t-test).

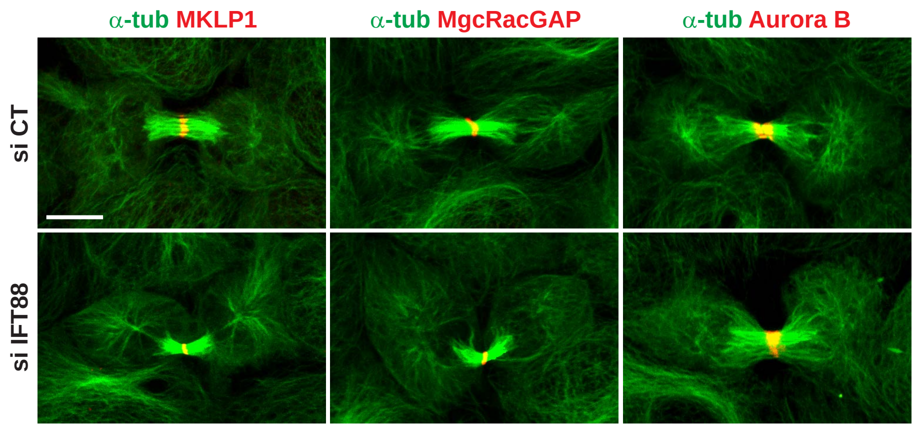

Supplementary Figure 7. MKLP1, MgcRacGAP and Aurora B are concentrated at the midbody at late stages of cytokinesis in both control and IFT88 depleted cells.
Immunofluorescence images showing MKLP1, MgcRacGAP and Aurora B accumulation at the midbody in LLC-PK1 cells transfected with CT or IFT88 siRNA. Scale bar: 10 µm.

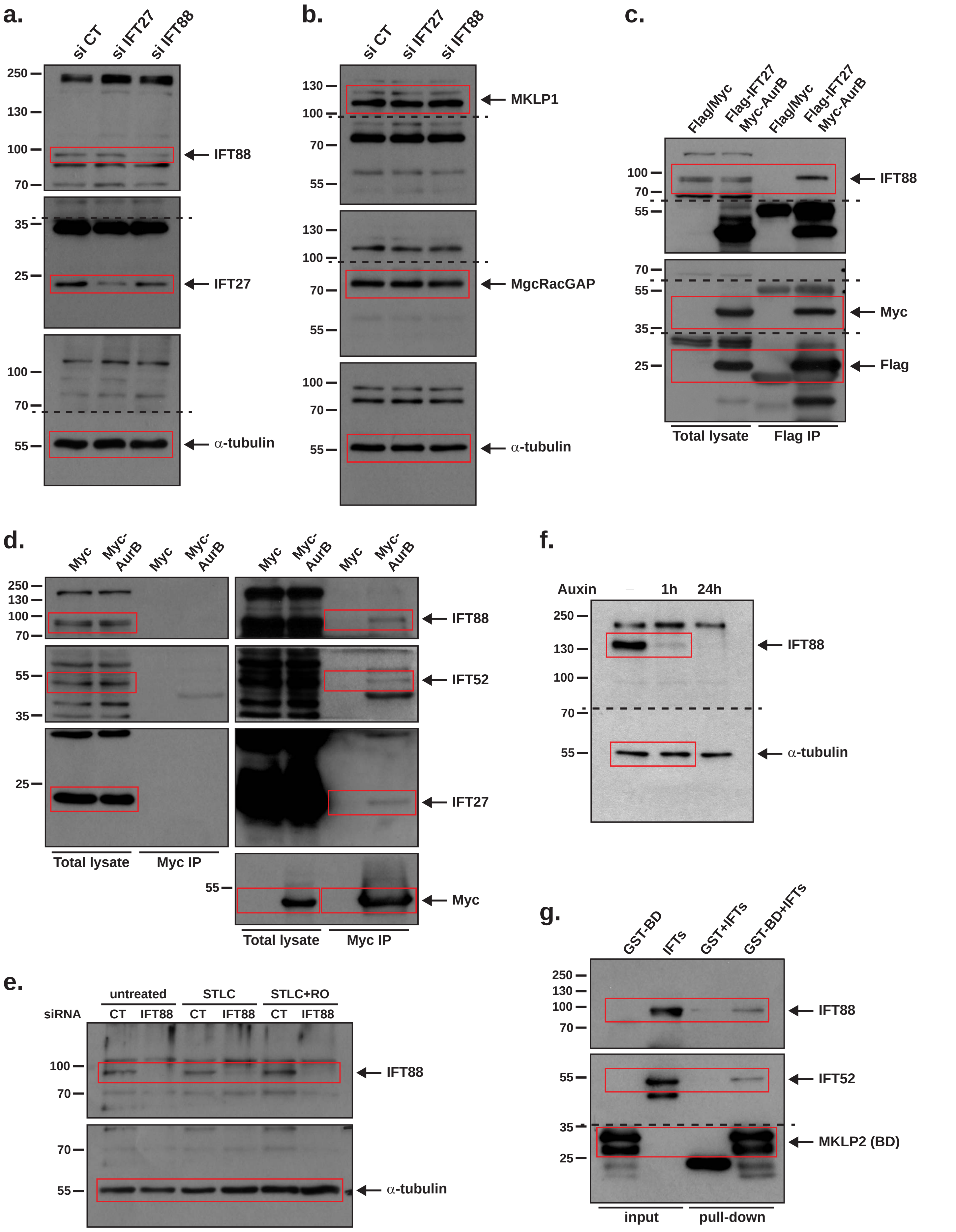

Supplementary Figure 8. Uncropped images for presented western-blots (Main figures)
(a) Corresponds to Figure 1b. **(b)** Corresponds to Figure 1i. **(c)** Corresponds to Figure 2a. **(d)** Corresponds to Figure 2b. **(e)** Corresponds to Figure 2g. **(f)** Corresponds to Figure 2j. **(g)** Corresponds to Figure 3b.

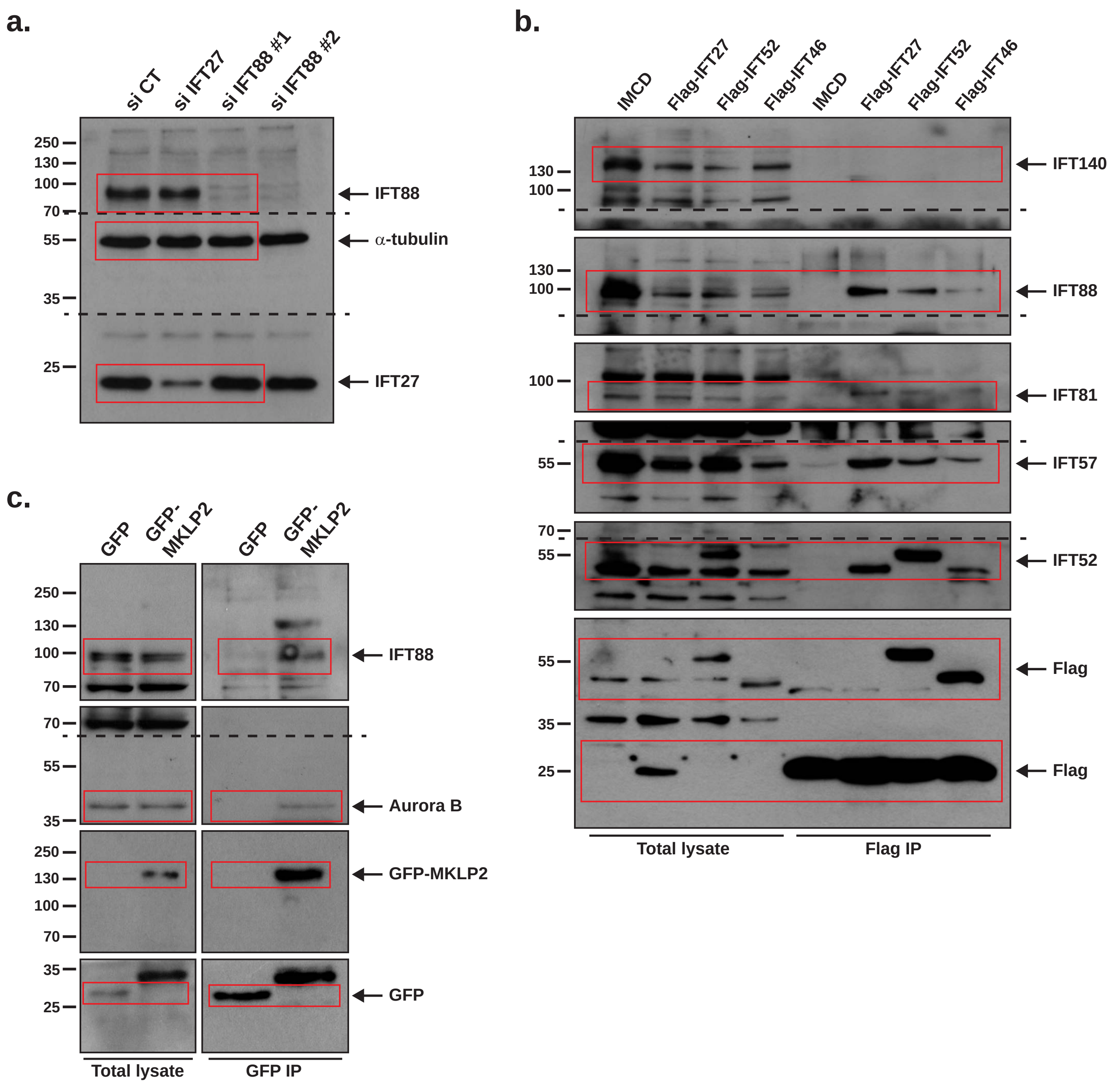

Supplementary Figure 9. Uncropped images for presented western-blots (Supplementary figures)
(a) Corresponds to Supplementary Figure 1c. **(b)** Corresponds to Supplementary Figure 2b. **(c)** Corresponds to Supplementary Figure 4a.

	IFT27-interacting proteins	% coverage
IFT-B complex	Intraflagellar transport protein 25	49,65
	Intraflagellar transport protein 27	49,46
	Intraflagellar transport protein 74	39,33
	Intraflagellar transport protein 22	38,92
	Intraflagellar transport protein 52	30,77
	Intraflagellar transport protein 81	28,99
	Intraflagellar transport protein 56	26,53
	Intraflagellar transport protein 38	22,76
	Intraflagellar transport protein 70	22,29
	Intraflagellar transport protein 46	16,94
	Intraflagellar transport protein 20	13,21
	Intraflagellar transport protein 57	12,62
	Intraflagellar transport protein 88	12,26
	Intraflagellar transport protein 80	6,06

Supplementary Table 1. A subset of IFT-B proteins interact in mitosis.
Summary of the proteomic analysis performed on IMCD cells stably expressing Flag-tagged IFT27 and synchronized in mitosis. Percentage of protein coverage is shown. Proteomic analysis led to the identification of other IFT-B complex proteins as IFT27 mitotic binding partners.
