# Supplementary material for: IFT proteins spatially control the geometry of cleavage furrow ingression and lumen positioning
Source: Nat Commun. 2017 Dec 4;8:1928. doi: 10.1038/s41467-017-01479-3 (PMC5715026; doi:10.1038/s41467-017-01479-3)
Supplement: Supplementary file 3 — Description of Additional Supplementary Files [file 41467_2017_1479_MOESM3_ESM.pdf]

Description of Additional Supplementary Files

File Name: Supplementary Movie 1
Description: Time-lapse microscopy of mitotic GFP-αtubulin LLC-PK1 cells transfected with CT or IFT88 siRNA. Time (min).

File Name: Supplementary Movie 2
Description: Time-lapse microscopy of mitotic GFP-MKLP2 HeLa Kyoto cells transfected with CT or IFT88 siRNA. Time (min).

File Name: Supplementary Movie 3
Description: Time-lapse microscopy showing cytokinesis progression of GFP-αtubulin/mCherry-H2B
LLC-PK1 cells transfected with CT or IFT88 siRNA. Time (min).

File Name: Supplementary Data 1
Description: Flag-IFT27 interactors identified by a mass-spectrometry approach performed on synchronized IMCD and IMCD Flag-IFT27 cells.
